# Supplementary material for: Coumarin and Moracin Derivatives from Mulberry Leaves (Morus alba L.) with Soluble Epoxide Hydrolase Inhibitory Activity
Source: Molecules. 2020 Aug 31;25(17):3967. doi: 10.3390/molecules25173967 (PMC7504814; doi:10.3390/molecules25173967)
Supplement: Supplementary file 1 [file molecules-25-03967-s001.pdf]

Supplementary Data

Coumarin and Moracin Derivatives from Mulberry Leaves (*Morus alba* L.) with Soluble Epoxide Hydrolase Inhibitory Activity

Hong Xu Li [1,2#], Myungsook Heo [4,#], Younghoon Go [3], Young Soo Kim [3], Young Ho Kim [4], Seo Young Yang [4,*], and Wei Li [3,*]

[1] College of Life Sciences and Oceanography, Shenzhen Key Laboratory of Marine Bioresource and Eco-environmental Science, Shenzhen University, Shenzhen 518060, China; charon0077@gmail.com

[2] College of Physics and Optoelectronic Engineering, Shenzhen University, Shenzhen 518060, China

[3] Korean Medicine (KM) Application Center, Korea Institute of Oriental Medicine, Daegu 41062, Republic of Korea; gotra827@kiom.re.kr (Y.H.G.); yskim527@kiom.re.kr (Y.S.K.); liwei1986@kiom.re.kr (W.L.)

[4] College of Pharmacy, Chungnam National University, Daejeon 34134, Republic of Korea; inyl1110@naver.com (M.H); syyang@cnu.ac.kr (S.Y.Y.); yhk@cnu.ac.kr (YH.K)

***** Correspondence: liwei1986@kiom.re.kr; Tel.: +82-53-940-3874 (W.L.) ; syyang@cnu.ac.kr; Tel.: +82-42-821-5933 (S.Y.Y.)

Figure S1. ^{1}H NMR spectrum of compound **1** in methanol-d_4 (300 MHz) ……………………………………………………………………...3

Figure S2. ^{13}C NMR spectrum of compound **1** in methanol-d_4 (75 MHz) ……………………………………………………………………...4

Figure S3. ^{1}H NMR spectrum of compound **2** in methanol-d_4 (600 MHz) ……………………………………………………………………...5

Figure S4. ^{13}C NMR spectrum of compound **2** in methanol-d_4 (150 MHz) …………………………………………………………………...6

Figure S5. ^{1}H NMR spectrum of compound **3** in dimethyl sulfoxide-d_6 (600 MHz) ……………………………………………………………...7

Figure S6. ^{13}C NMR spectrum of compound **3** in dimethyl sulfoxide-d_6 (150 MHz) …………………………………………………………...8

Figure S7. ^{1}H NMR spectrum of compound **4** in methanol-d_4 (600 MHz) ……………………………………………………………………...9

Figure S8. ^{13}C NMR spectrum of compound **4** in methanol-d_4 (150 MHz) …………………………………………………………………...10

Figure S9. ^{1}H NMR spectrum of compound **5** in methanol-d_4 (600 MHz) …………………………………………………………………...11

Figure S10. ^{13}C NMR spectrum of compound **5** in methanol-d_4 (150 MHz) ………………………………………………………………...12

Figure S11. ^{1}H NMR spectrum of compound **6** in methanol-d_4 (600 MHz) …………………………………………………………………...13

Figure S12. ^{13}C NMR spectrum of compound **6** in methanol-d_4 (150 MHz) ………………………………………………………………...14

Figure S13. ^{1}H NMR spectrum of compound **7** in methanol-d_4 (600 MHz) …………………………………………………………………...15

Figure S14. ^{13}C NMR spectrum of compound **7** in methanol-d_4 (150 MHz) ………………………………………………………………...16

Figure S15. ^{1}H NMR spectrum of compound **8** in methanol-d_4 (600 MHz) …………………………………………………………………...17

Figure S16. ^{13}C NMR spectrum of compound **8** in methanol-d_4 (150 MHz) ………………………………………………………………...18

Figure S17. ^{1}H NMR spectrum of compound **9** in dimethyl sulfoxide-d_6 (600 MHz) …………………………………………………………...19

Figure S18. ^{13}C NMR spectrum of compound **9** in dimethyl sulfoxide-d_6 (150 MHz) ………………………………………………………...20

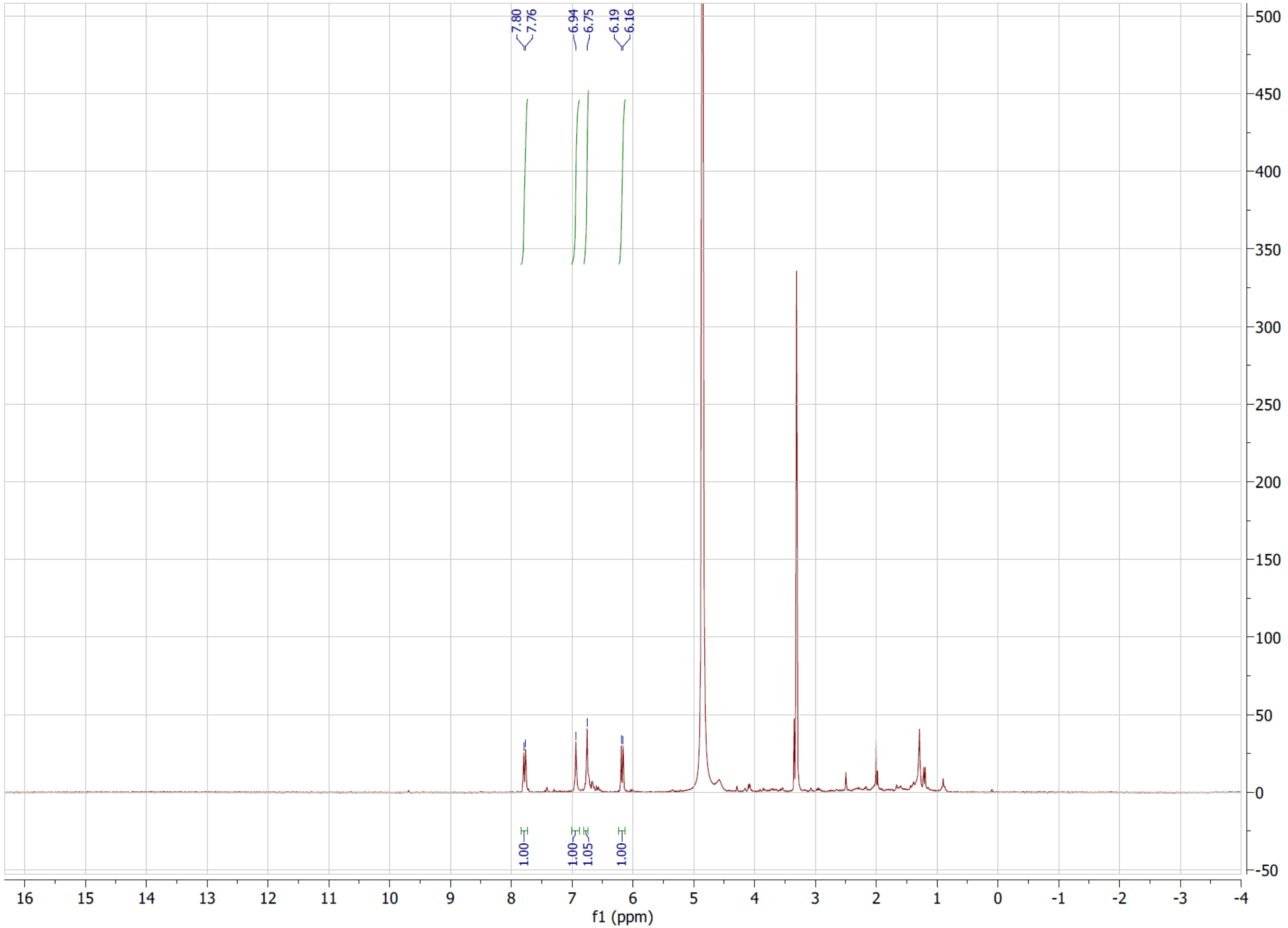

Figure S1. ^{1}H NMR spectrum of compound **1** in methanol-d_4 (300 MHz)

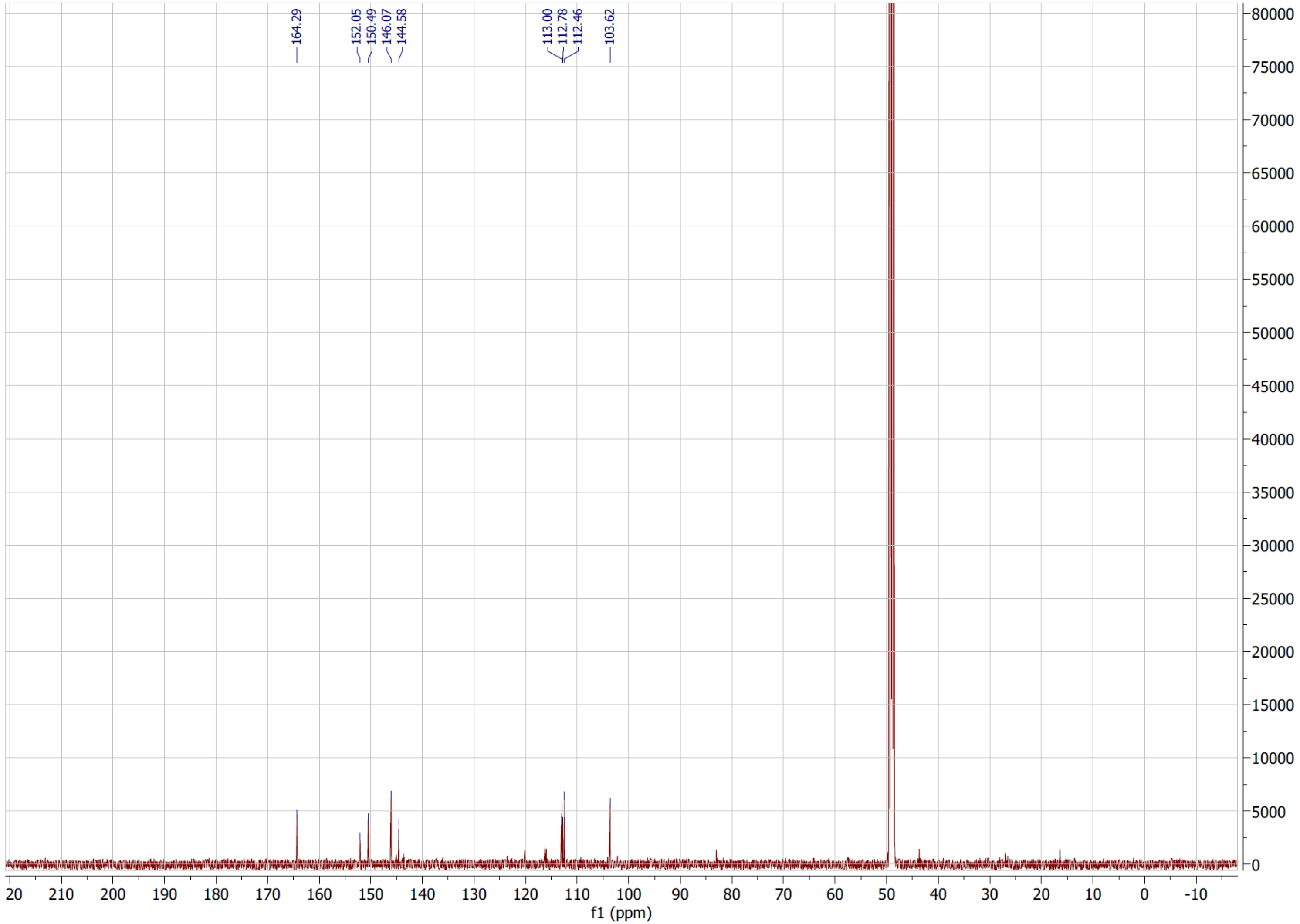

Figure S2. ^{13}C NMR spectrum of compound **1** in methanol-d_4 (75 MHz)

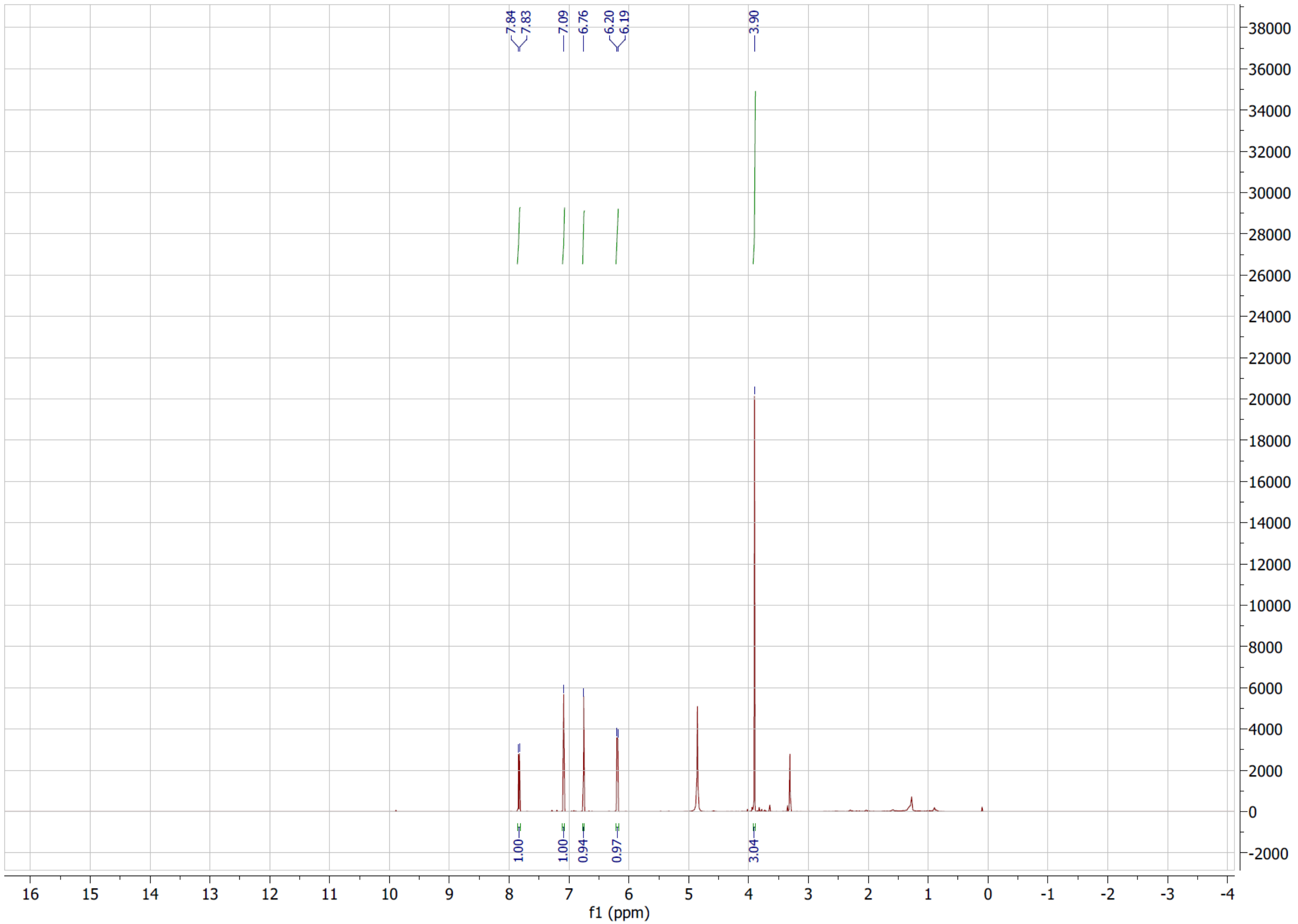

Figure S3. ^{1}H NMR spectrum of compound **2** in methanol-d_4 (600 MHz)

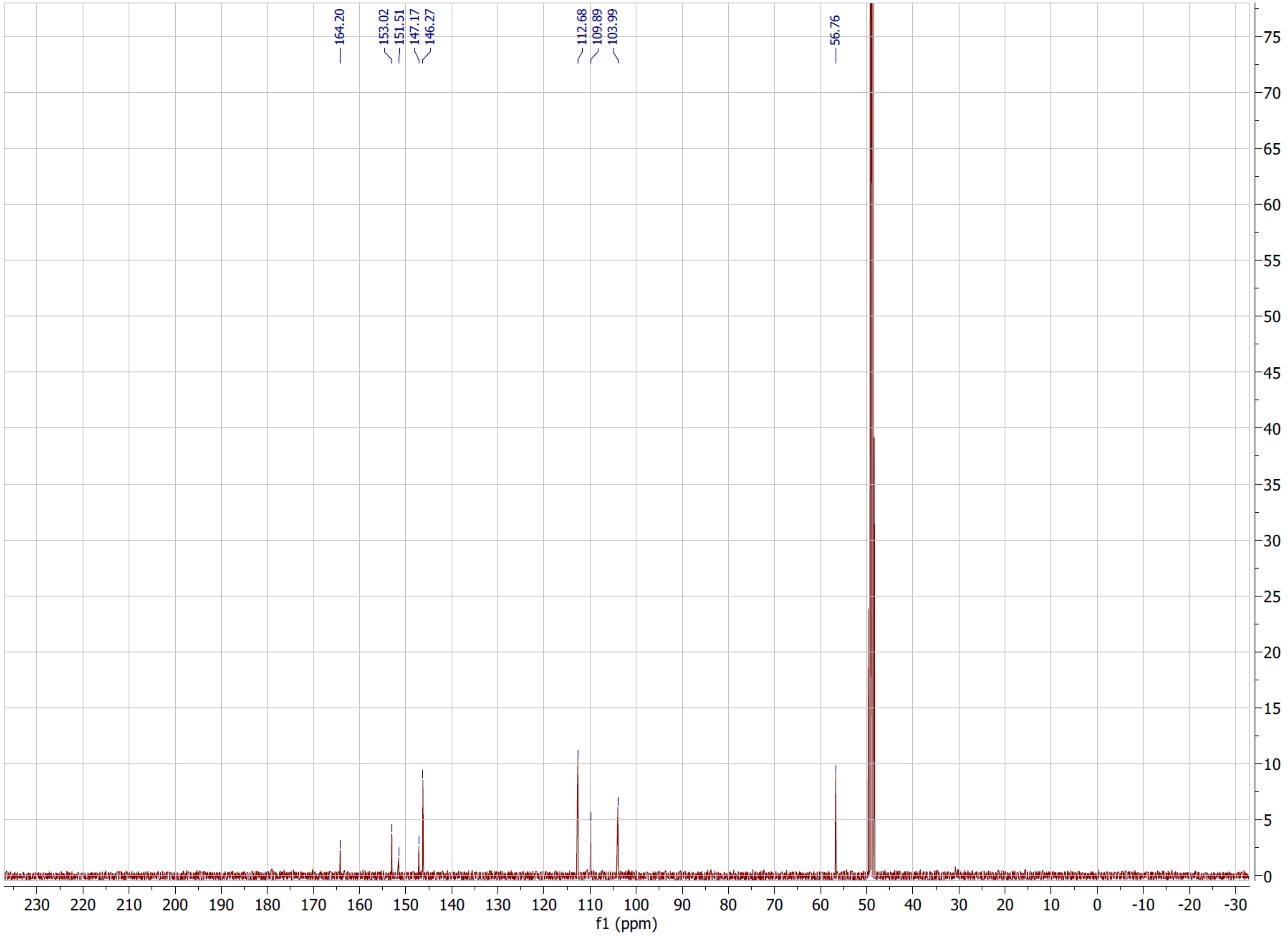

Figure S4. ^{13}C NMR spectrum of compound **2** in methanol-d_4 (150 MHz)

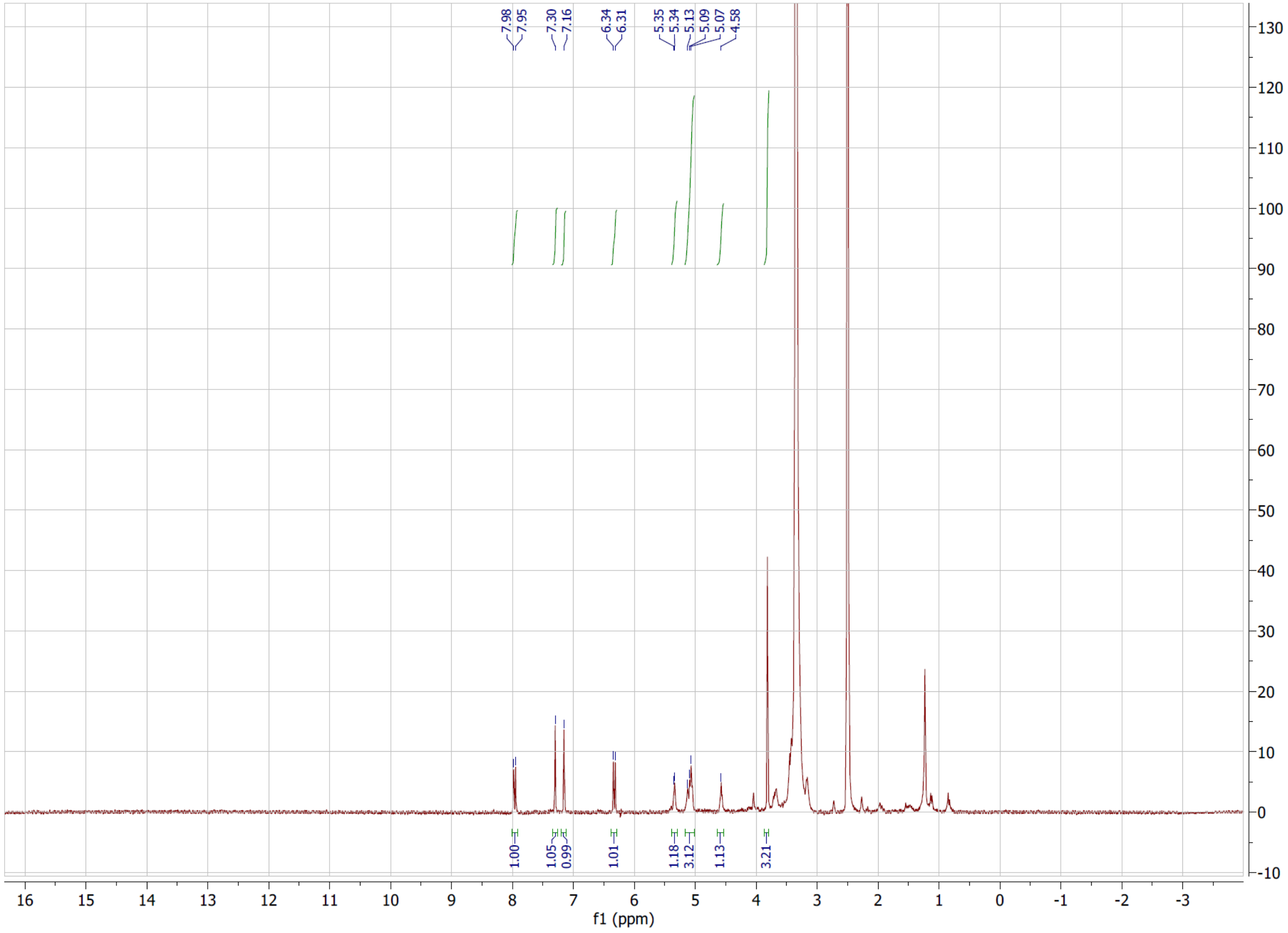

Figure S5. ^{1}H NMR spectrum of compound **3** in dimethyl sulfoxide-d_6 (600 MHz)

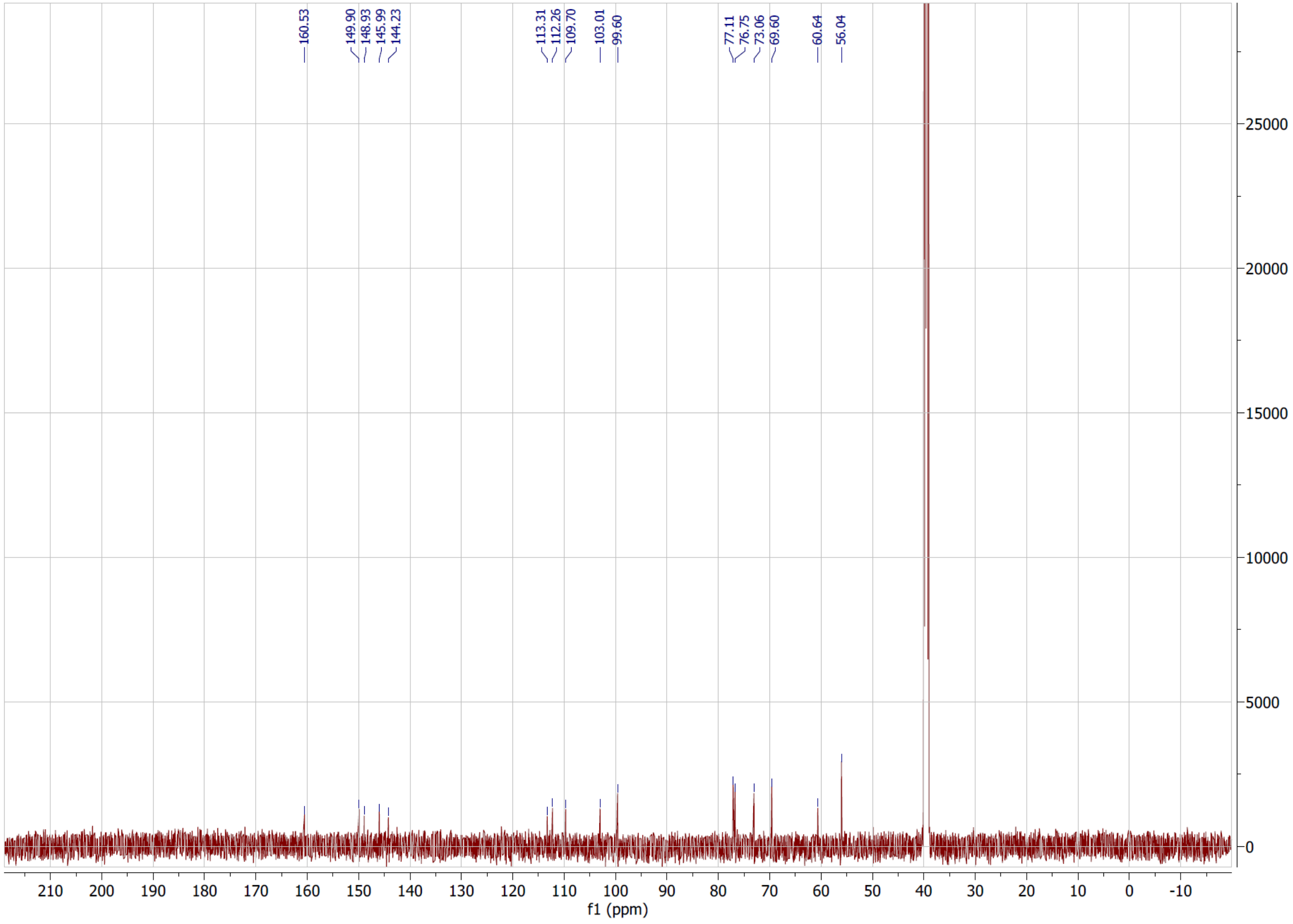

Figure S6. ^{13}C NMR spectrum of compound **3** in dimethyl sulfoxide-d_6 (150 MHz)

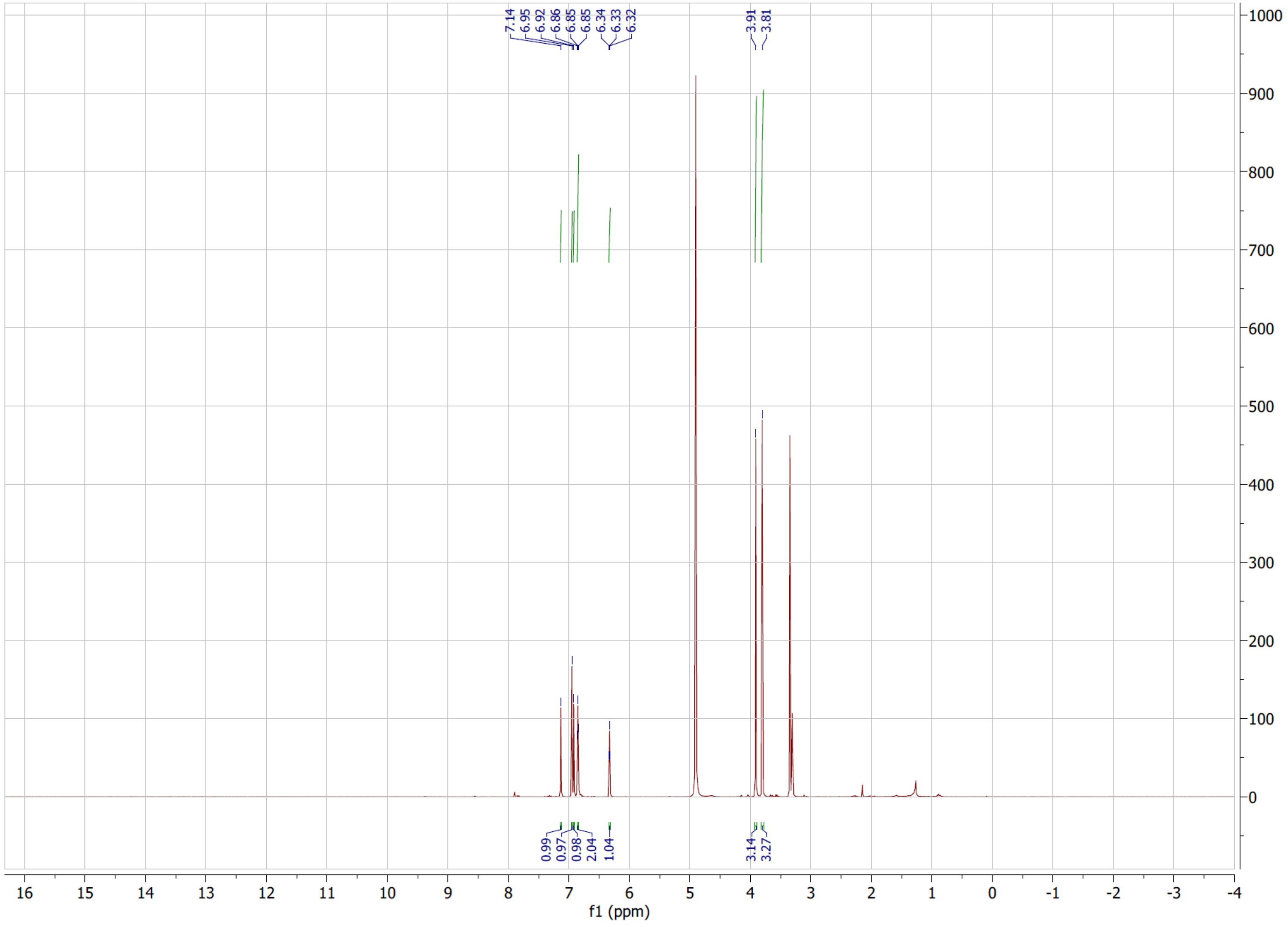

Figure S7. ^{1}H NMR spectrum of compound **4** in methanol-d_4 (600 MHz)

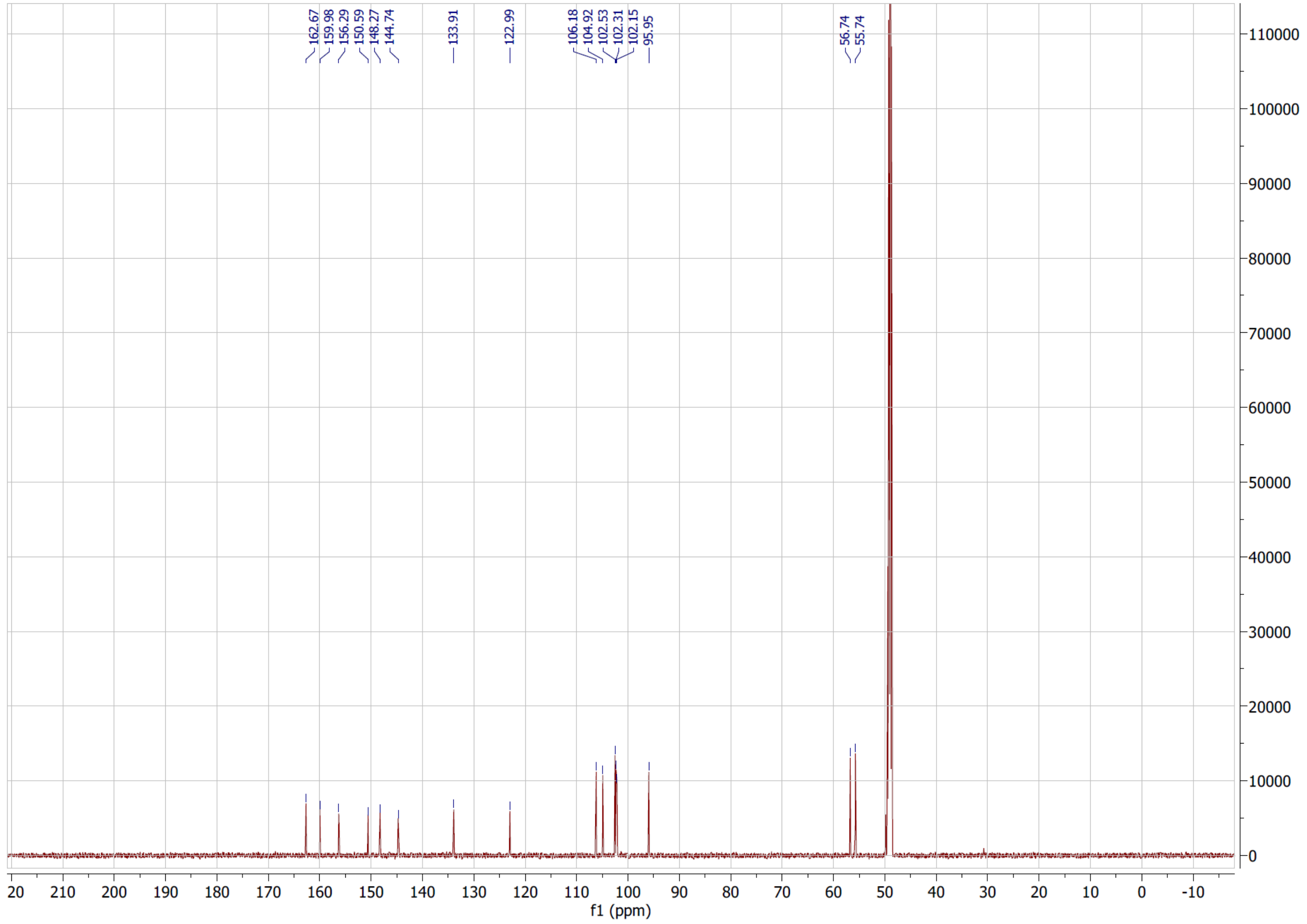

Figure S8. ^{13}C NMR spectrum of compound **4** in methanol-d_4 (150 MHz)

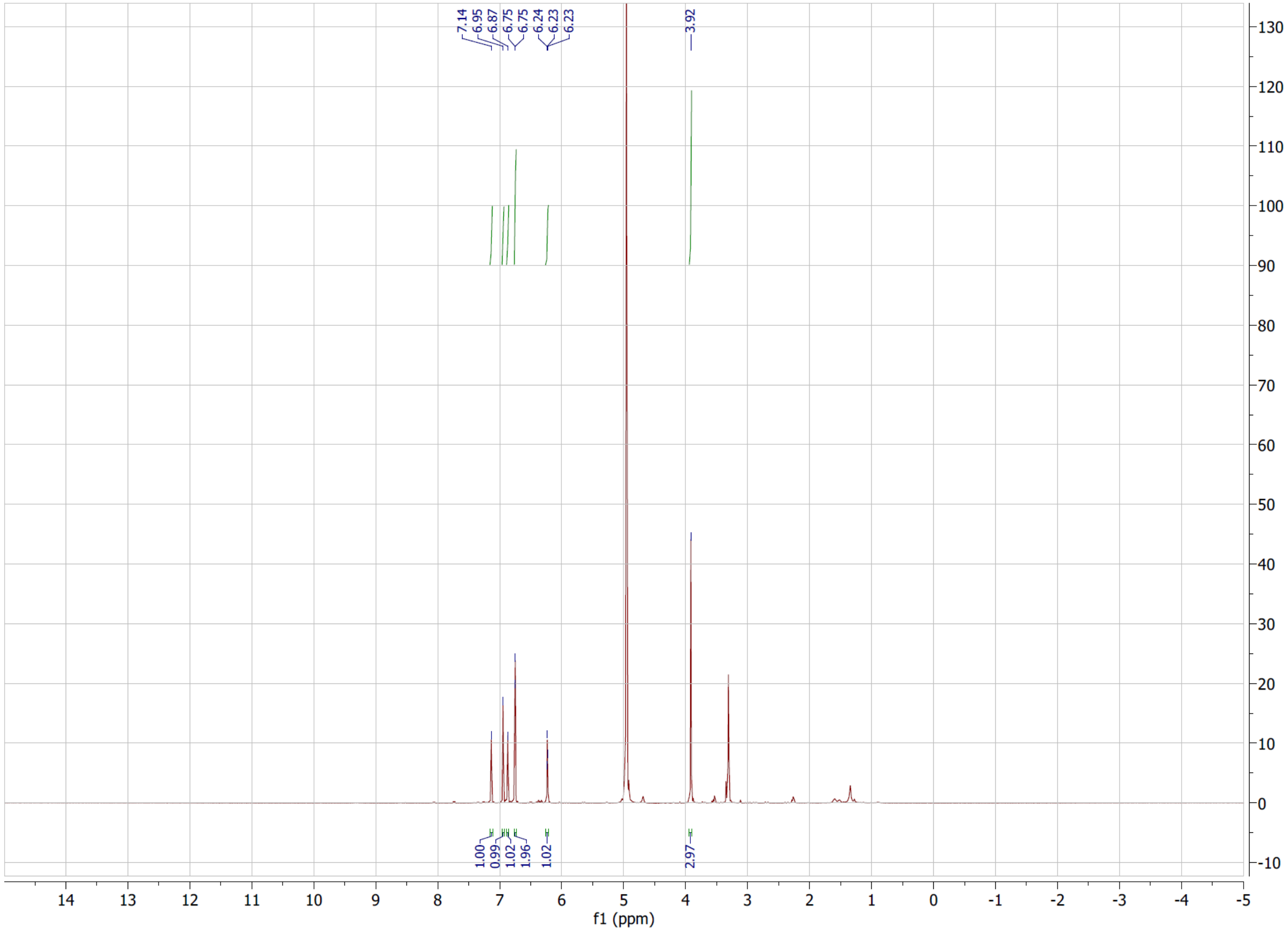

Figure S9. ^{1}H NMR spectrum of compound **5** in methanol-d_4 (600 MHz)

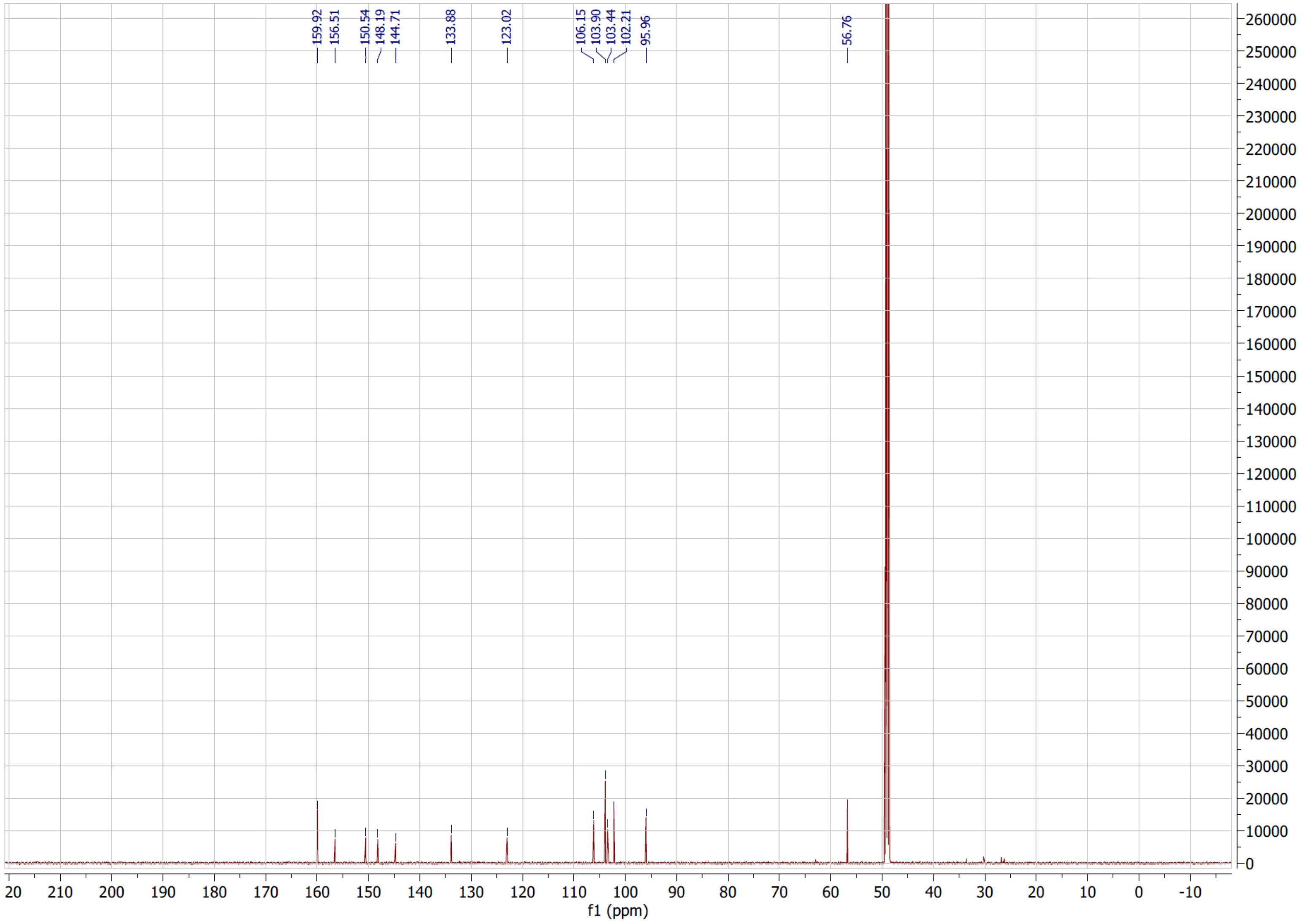

Figure S10. ^{13}C NMR spectrum of compound **5** in methanol-d_4 (150 MHz)

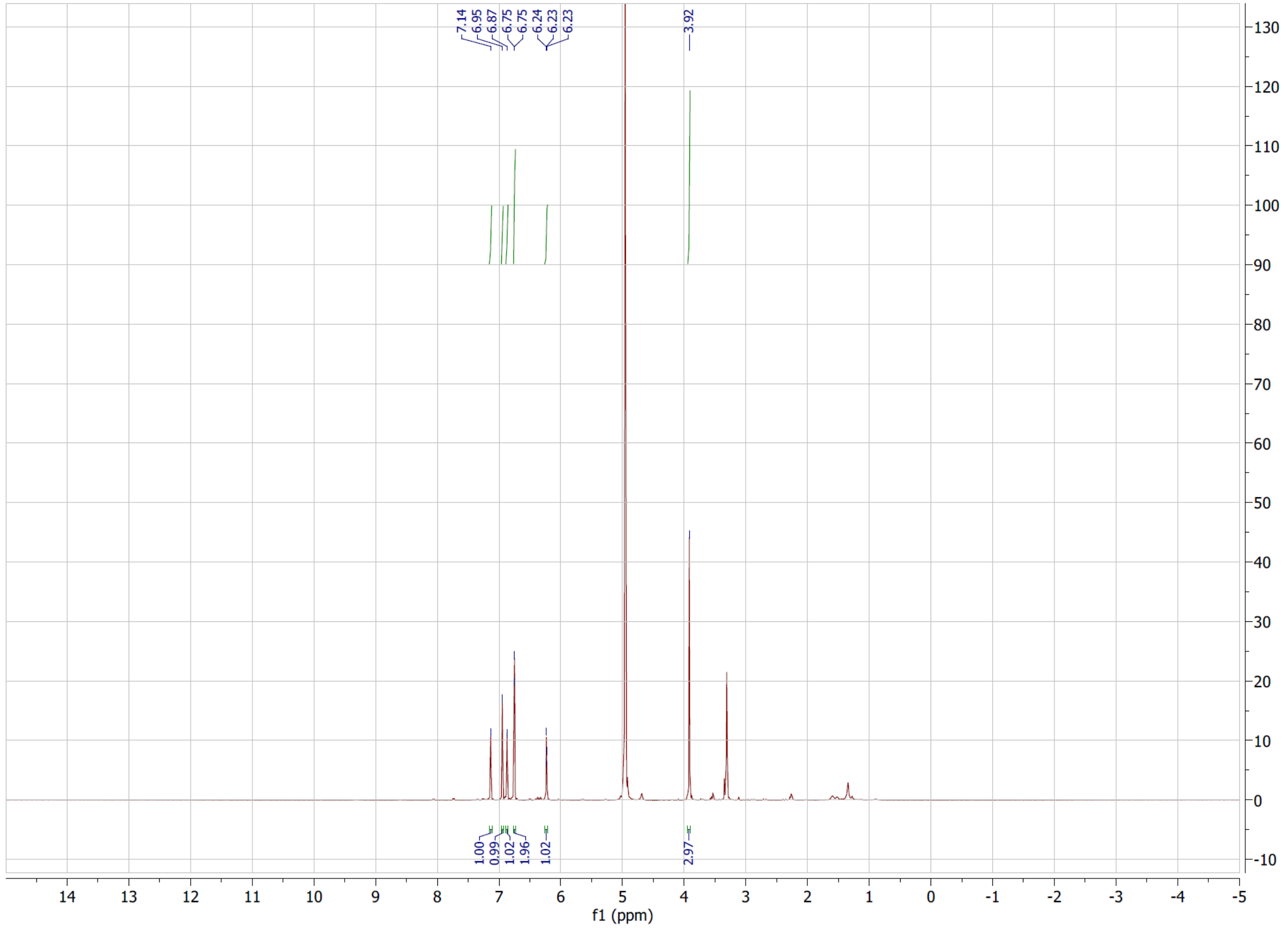

Figure S11. ^{1}H NMR spectrum of compound **6** in methanol-d_4 (600 MHz)

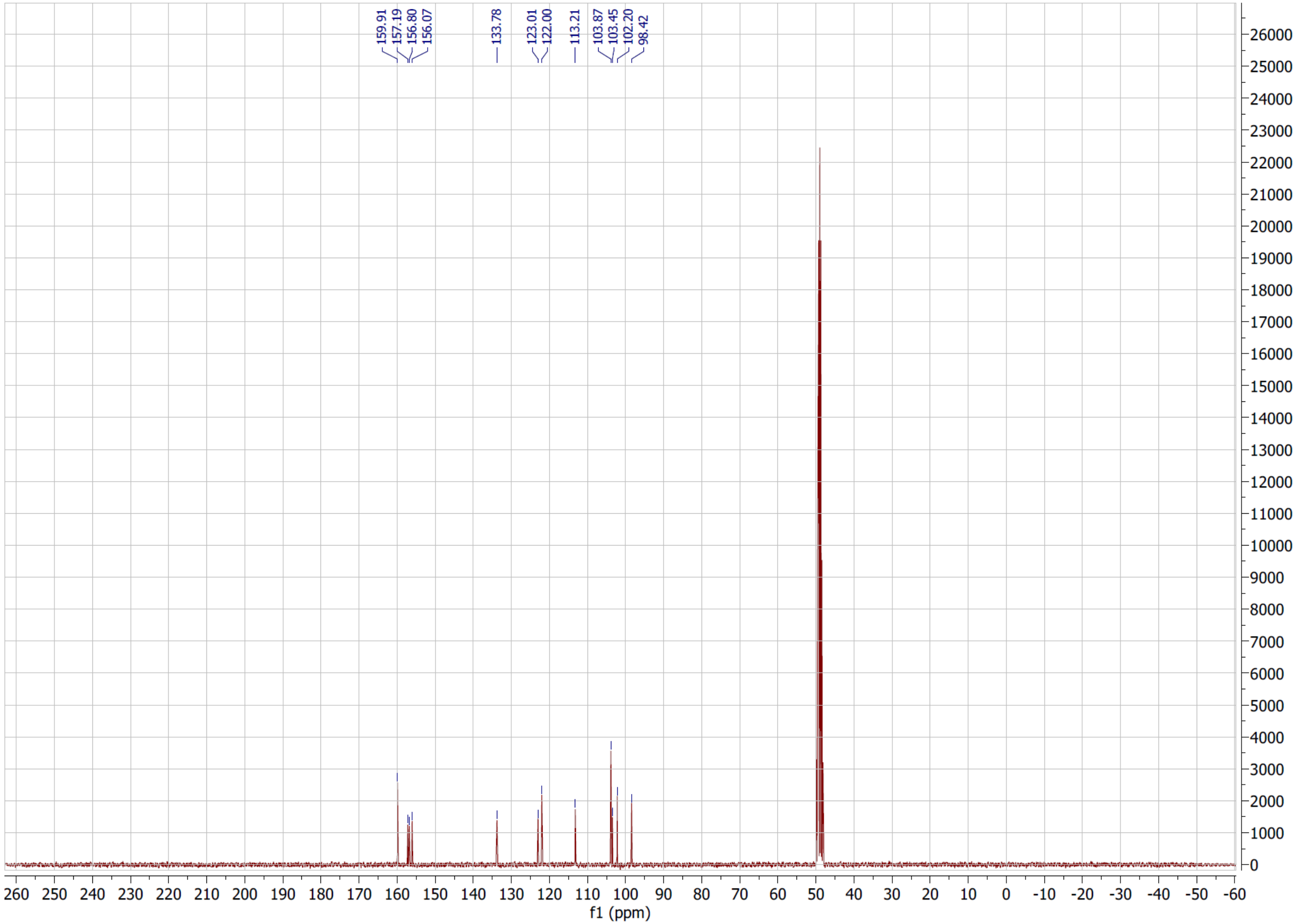

Figure S12. ^{13}C NMR spectrum of compound **6** in methanol-d_4 (150 MHz)

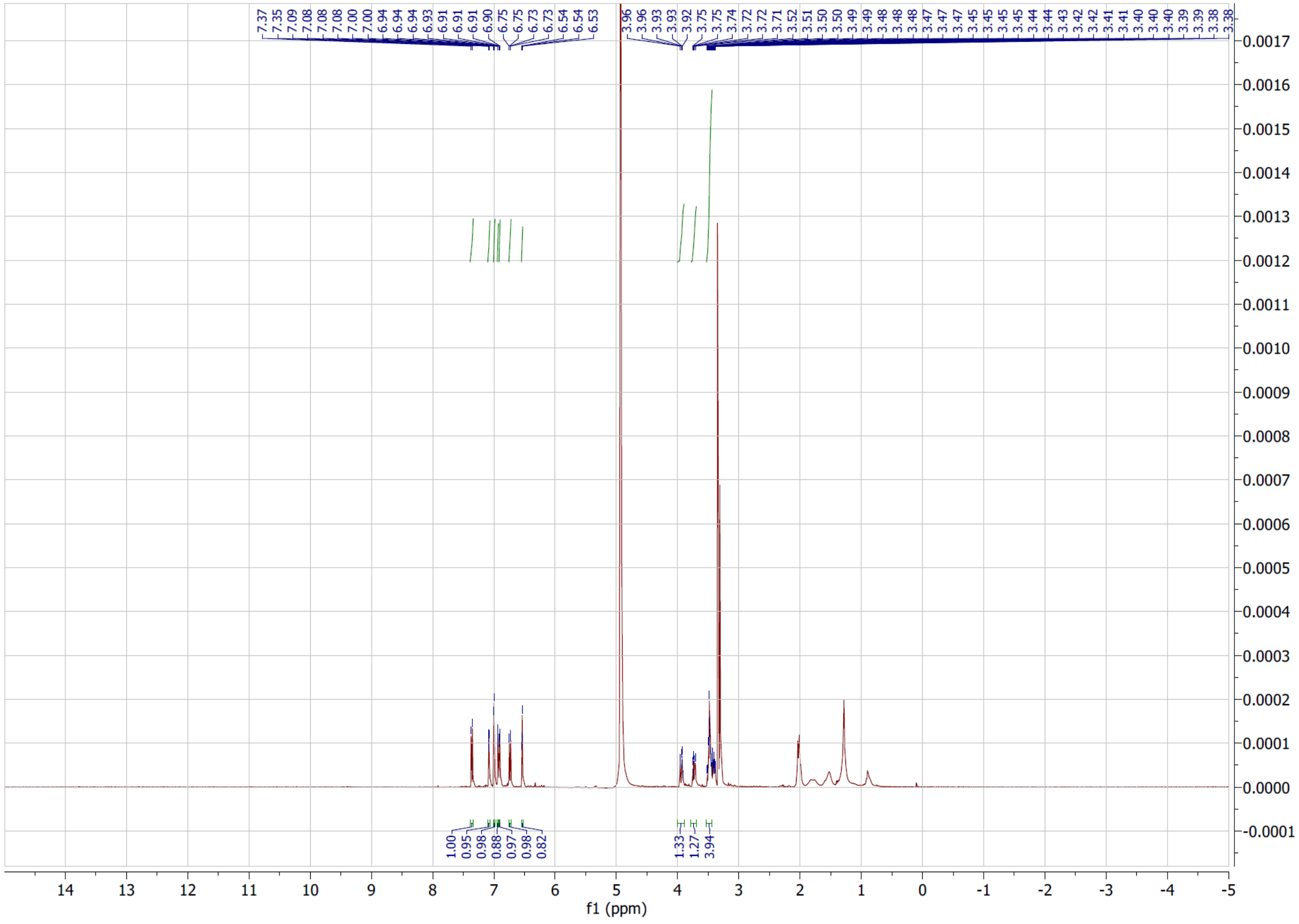

Figure S13. ^{1}H NMR spectrum of compound **7** in methanol-d_4 (600 MHz)

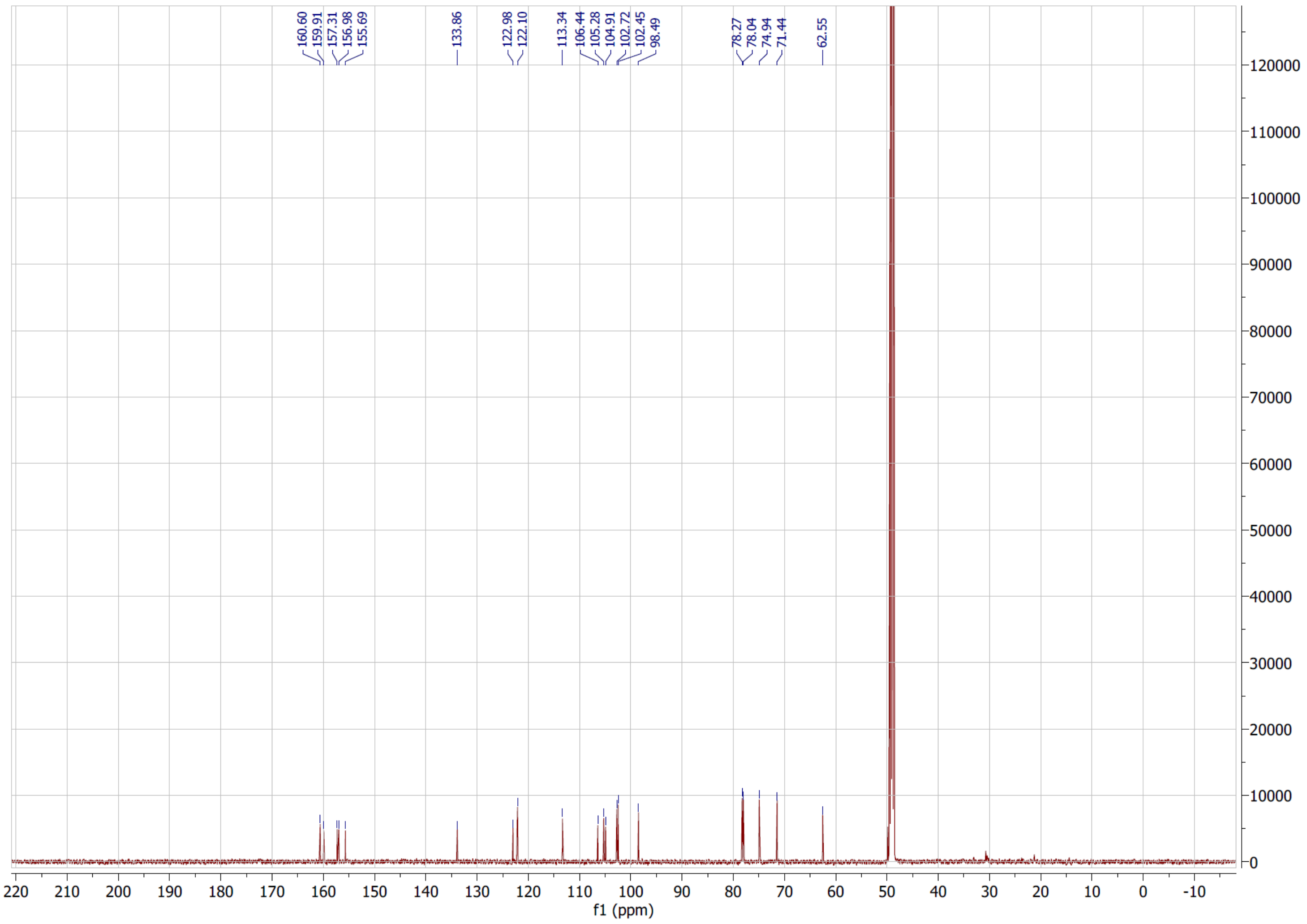

Figure S14. ^{13}C NMR spectrum of compound **7** in methanol-d_4 (150 MHz)

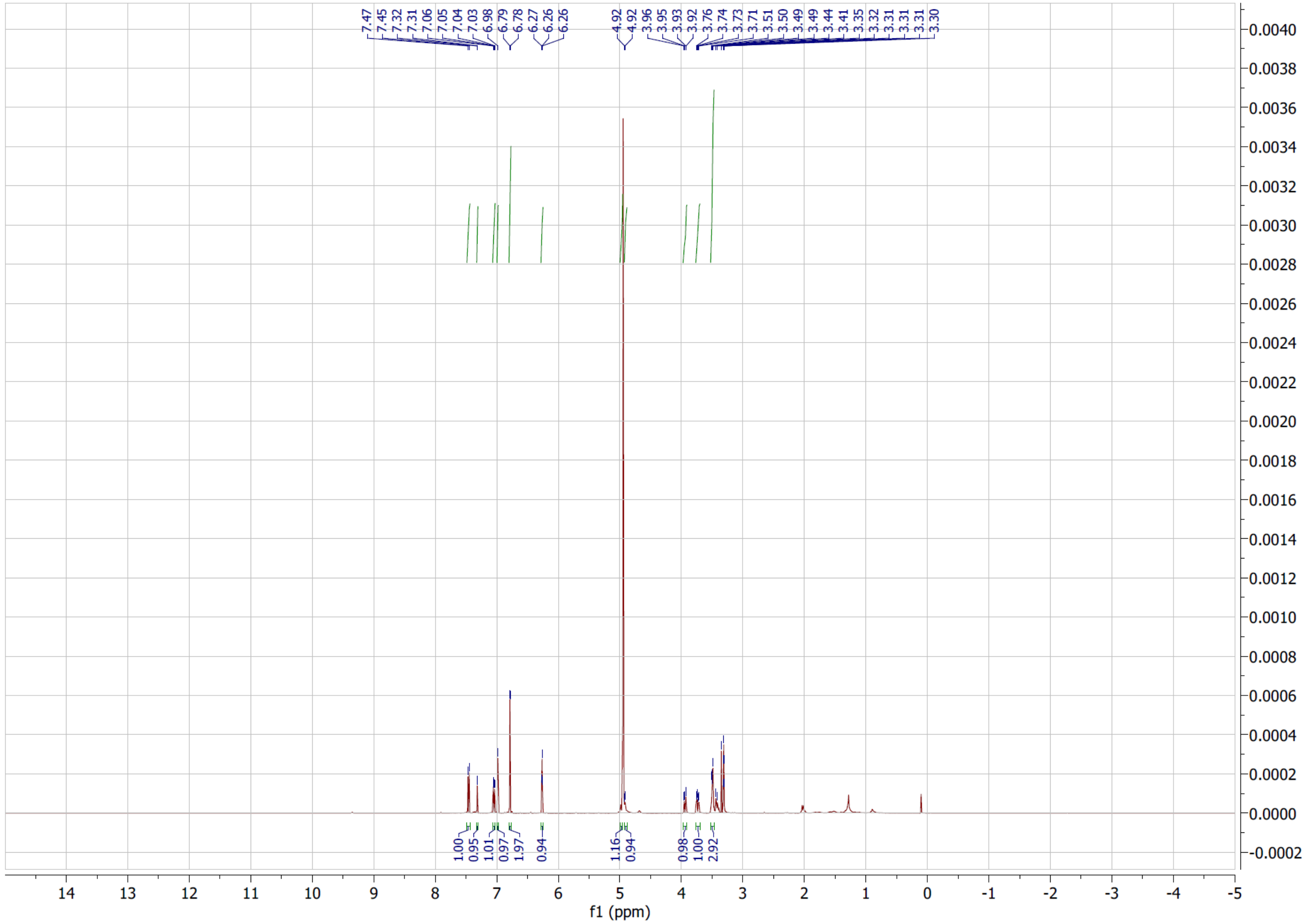

Figure S15. ^{1}H NMR spectrum of compound **8** in methanol-d_4 (600 MHz)

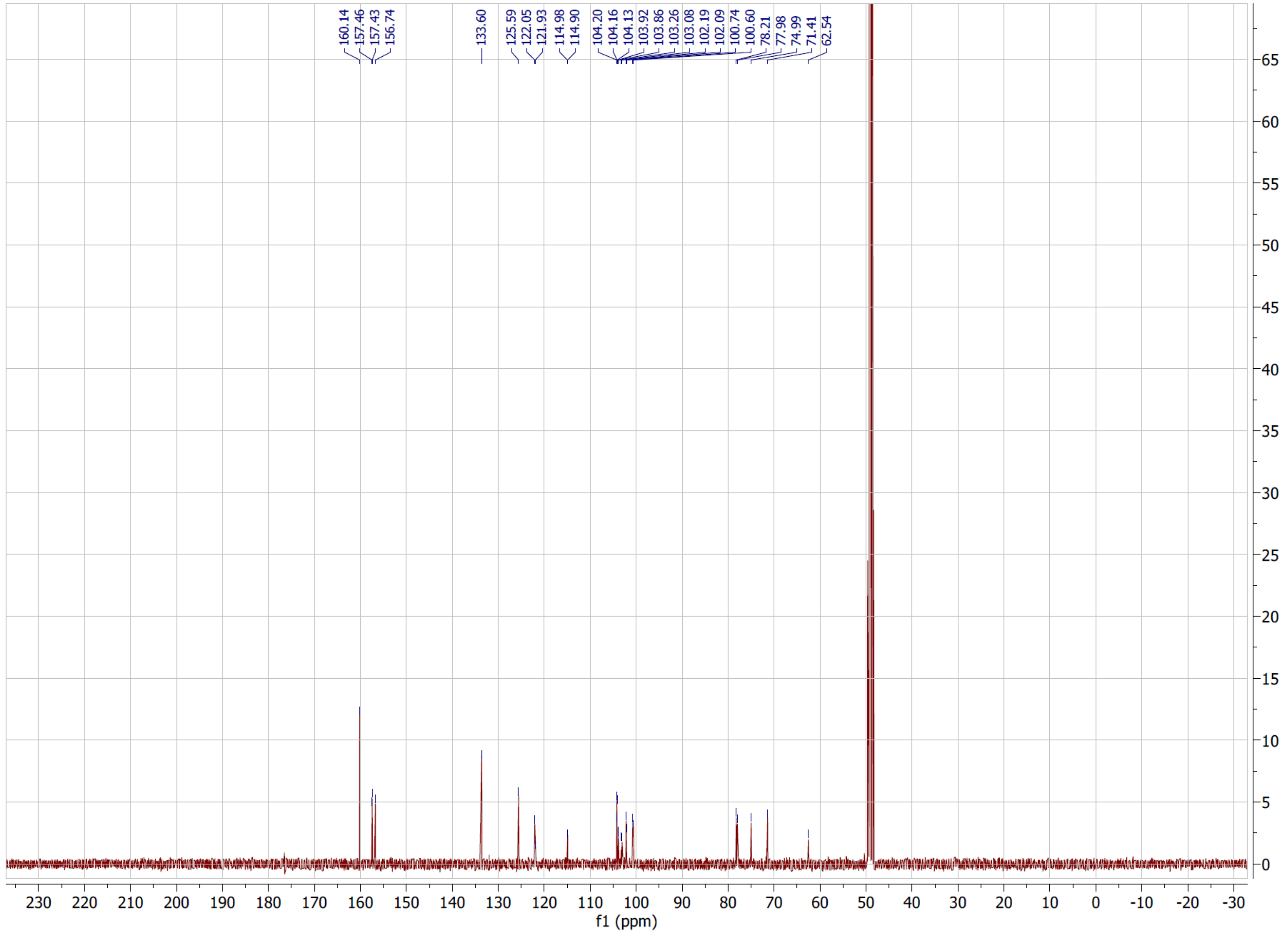

Figure S16. ^{13}C NMR spectrum of compound **8** in methanol-d_4 (150 MHz)

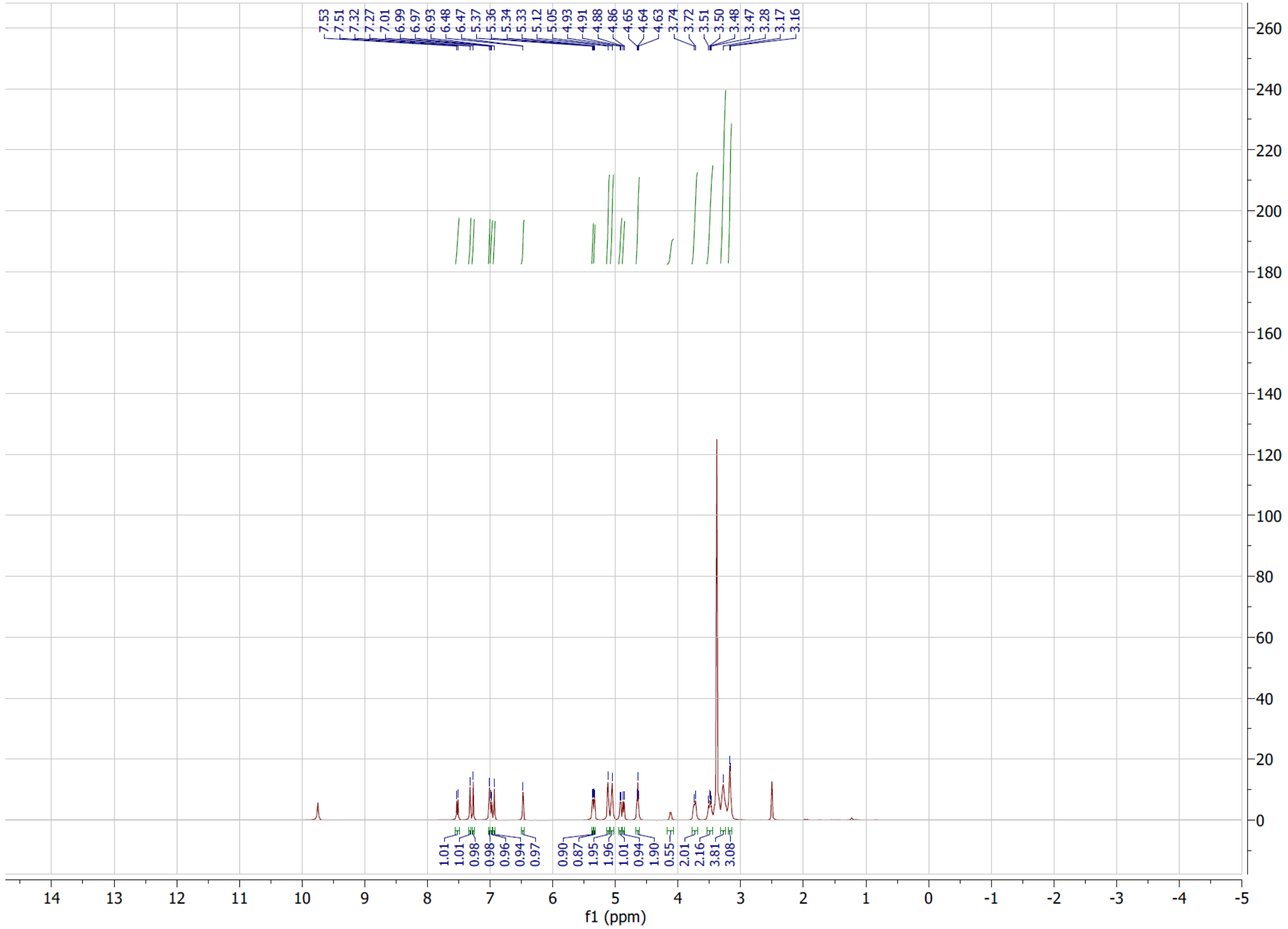

Figure S17. ^{1}H NMR spectrum of compound **9** in dimethyl sulfoxide-d_6 (600 MHz)

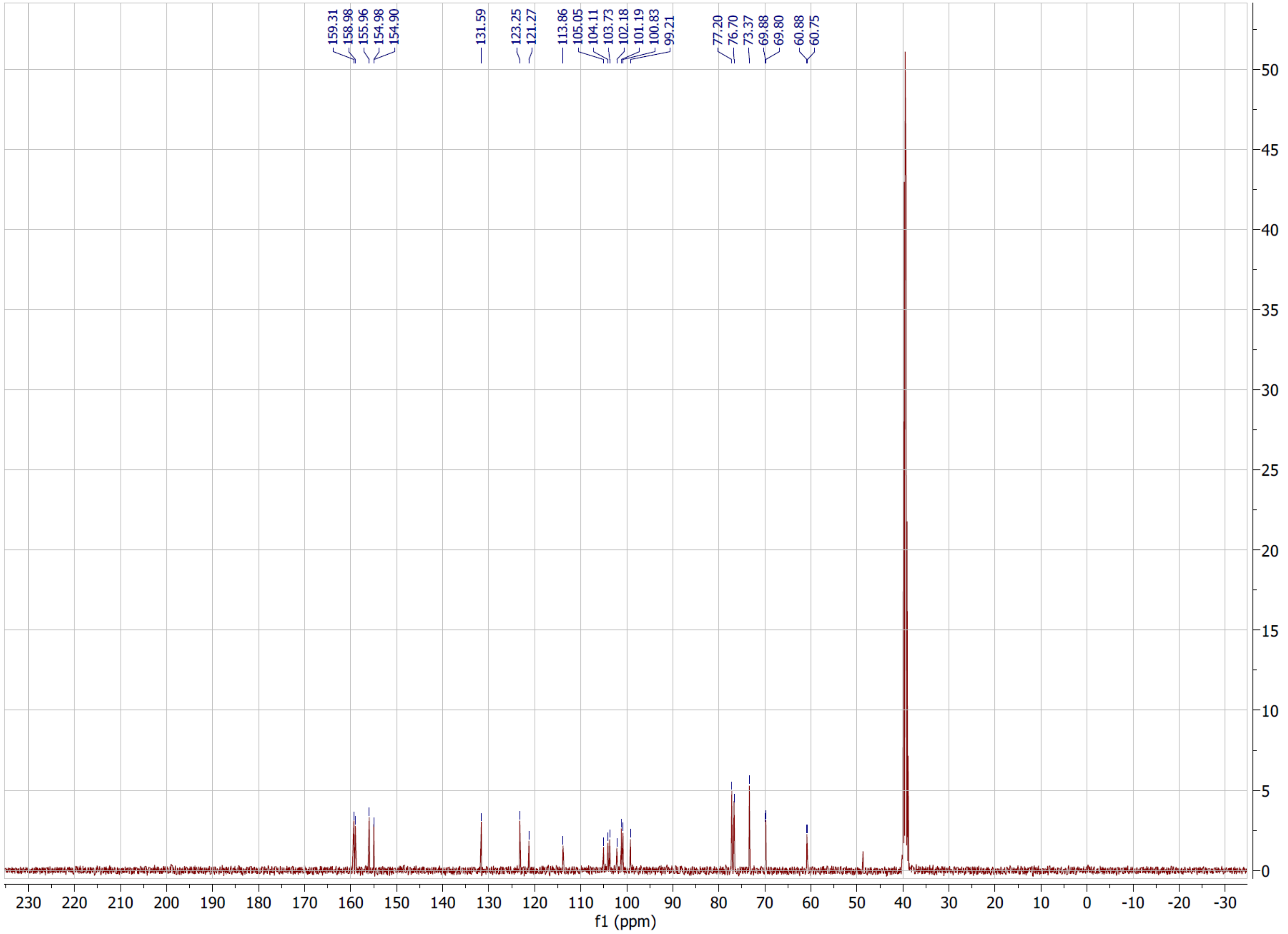

Figure S18. ^{13}C NMR spectrum of compound **9** in dimethyl sulfoxide-d_6 (150 MHz)
